# Association between depression, anxiety, stress and perceived quality of life in a Malaysian B40 urban community during the COVID-19 lockdown: A cross-sectional study

**DOI:** 10.12688/f1000research.51924.1

**Published:** 2021-07-29

**Authors:** Gan Sing Joo, Daniel Mahalingam Owen Devan, Chuah Shao Qi, Sapna Shridhar Patil

**Affiliations:** 1 School of Medicine, Faculty of Health and Medical Sciences, Taylor's University, Subang Jaya, Selangor, 47500, Malaysia

**Keywords:** Malaysian B40 community, Quality of Life, DASS-21, WHOQOL-BREF, Association, COVID-19 pandemic, lockdown

## Abstract

**Background:** In Malaysia, B40 communities are those with a median monthly household earning of under RM 3166. With the prolonged COVID-19 pandemic and the resulting lockdown, the livelihoods of those in these areas has been severely impacted. This has increased their vulnerability to psychological afflictions and has led to a poorer perception of their quality of life (QoL) in comparison to the rest of the population. We investigated the association between perceived levels of depression, anxiety and stress and their impact on quality of life (QoL) among B40 residents in a low-cost urban housing area in Kuala Lumpur, Malaysia.

**Methods: **A cross-sectional study was conducted between July 2020 and February 2021 in the Seri Pantai housing settlement. The validated Malay versions of the depression, anxiety, and stress scale-21 (DASS-21) and the World Health Organization Quality of Life, brief (WHOQOL-BREF) were distributed to the participants using Google forms. The statistical significance of the association between subscales of depression, anxiety, stress and QoL domains were assessed using the Pearson’s correlation test.

**Results:** Of the 180 participants, the majority were Malays (87.2%) and females (82.2%). The average scores were the highest for stress (5.66 ± 4.59) and the score in the environment domain of QoL (59.27 ± 17.23) was the lowest. A statistically significant negative correlation was found between the subscales of DASS-21 and the four domains of the QoL, with the social relationships and psychological domains showing a highly significant association (p < 0.001). The strongest correlation was observed between the psychological domain and depression (r= -.520) followed by psychological domain and stress (r= -.496).

**Conclusion:** The strongest correlation was observed between psychological domain and depression. This suggests a need to address potential devastating mental health consequences of the COVID-19 pandemic and its effect on the QoL of residents in B40 communities.

## Introduction

Quality of life (QoL) is defined as an individual's perception of their position in life in the context of the culture and value systems in which they live and in relation to their goals, expectations, standards and concerns ( World Health Organization Quality of Life [WHOQOL], 2012). This humanistic element was introduced to expand the focus of measuring health holistically beyond the norm of mortality and morbidity ( WHOQOL, 1994). Globally, there are more than 150 measures available which are used to assess perceived quality of life ( Gill, 1994). This includes the Short Form-36 Health Survey ( Rand, 1980), The Quality-of-Life Scale (Flanagan, 1978) and EuroQoL-5D or EQ-5D ( Devlin & Brooks, 2017). However, the tool used most worldwide is the WHOQOL questionnaire - the WHOQOL-100 being the original complete version and the WHOQOL-BREF assessment being the abbreviated version, which are applicable cross-culturally and translated accordingly, and include translation to the Malay language ( Cheung *et al.*, 2019; Hasanah *et al*., 2003; Iqbal *et al.*, 2020).

The Department of Statistics Malaysia ( DOSM, 2020) categorized the Malaysian population into T20 (top 20), M40 (middle 40) and B40 (bottom 40) according to monthly household income. B40 communities are those with monthly household earnings of under RM 4850 and a mean monthly income of RM 3166.

Perceived QoL has been shown to differ between various communities and is affected by several factors such as income stability, health status, and socioeconomic status ( Bielderman *et al.*, 2015; Cruz *et al.*, 2011; Khan & Tahir, 2014; Shahar *et al.*, 2019; Wan Puteh *et al.*, 2019). Locally, a study done by Wan Puteh *et al.* in 2019 found that statistically significant low QoL was observed in single, male, low-income households, and in households with chronic illness. These results concurred with those reported by Cruz *et al.* (2011) and Skevington and McCrate (2012).

Poor mental health is associated with low perceived QoL ( Bujang *et al.*, 2015; Clavecillas & Perez, 2020; Farris *et al*., 2020; Gan & Hue, 2019; Tan and Yadav, 2013). Lower socioeconomic groups are two to three times more likely to suffer from mental health issues than those in higher socioeconomic groups ( Kim & Cho, 2020). Meta-analysis by Lorant *et al.* (2003) also revealed significant evidence of the correlation between socioeconomic inequality and depression. Income inequality during rapid urbanization in Malaysia has led to the urban poor being more susceptible to mental health disorders than the others. The DASS-21 (Depression, Anxiety and Stress Scale - 21 items) questionnaire has been proven to be reliable and valid in the context of Malaysians, and was used to assess the mental health of the participants in this study ( Musa *et al.*, 2007; Oei *et al.*, 2013; Ramli *et al.*, 2009).

The current COVID-19 lockdown has led to a substantial increase in symptoms of depression, anxiety, and stress among the general population ( Farris *et al.*, 2020; Patel *et al*., 2020). Income loss, social isolation, and poor physical health were shown to be factors which exacerbated mental ill-health ( Pfefferbaum & North, 2020). In a study conducted mid-pandemic, Ali *et al.* (2020) assessed the QoL and levels of DAS (Depression, Anxiety, Stress) in the general population of India. Out of 847 participants, 61.9% were found to have psychological symptoms of DAS, and 36.1% were reported having poor QoL after 45 days of lockdown.

In Malaysia, Gan and Hue (2019) investigated the levels of anxiety, depression and QoL among medical students using the Hospital Anxiety and Depression Scale (HADS) and WHOQOL-BREF. Among 149 medical students, the prevalence rates of anxiety and depression were 33% and 11% respectively. Anxiety was significantly associated with lower psychological, social, and environmental scores, while depression was significantly associated with lower physical, psychological, and environmental scores.

Recently, a sample of 326 participants was recruited from several urban communities in Malaysia and participants were assigned the DASS-21 and WHOQOL-BREF ( Farris *et al.*, 2020). Similar to previous studies by Shamsuddin *et al.* (2013) and Aziella *et al.* (2017), it was shown that anxiety had the highest prevalence (41.7%) among the participants. The sample was only drawn from urban communities because the authors presumed a higher prevalence of mental issues in urban settings ( Farris *et al*., 2020). This supports Gruebner *et al.* (2017) who proved that in the urban population, there are higher odds of developing mood disorders (1.4-fold) and anxiety disorders (1.2 fold) than in the rural population.

The academic community has extensively explored the relationship between perceived QoL and DASS-21 ( Ali *et al.*, 2020; Clavecillas & Perez, 2020; Farris *et al.*, 2020; Patil, 2021). However, studies on the association between DAS and QoL are still scarce in the international literature and few studies have been conducted among the B40 community in Malaysia ( Bujang *et al.*, 2015; Gan & Hue, 2019). Moreover, the study of these associations is of particular importance during the ongoing COVID-19 pandemic. Understanding the association between DAS and QoL may shed light on the need for future policies relating to the welfare of the urban poor. Thus, the aim of this research is to investigate the association between DAS and QoL in a low socioeconomic community in a developing country during the pandemic lockdown.

## Methods

### Study design and setting

This cross-sectional study was conducted among the inhabitants of the Seri Pantai Community Housing Program, also known as Program Perumahan Rakyat (PPR) in Kuala Lumpur, Malaysia. The PPR is a community housing project which was developed by the National Housing Department (Jabatan Perumahan Negara) in 1998 in an effort to improve the housing environment of the low income (B40) urban dwellers ( Hami Erina, 2013). The low-cost, high-rise apartments of Seri Pantai PPR comprises two, 21-storey blocks with approximately 20 households per floor.

### Participant recruitment and data collection

The recruitment of participants and data collection took place from July 2020 to February 2021 with the help of community leaders. These community leaders are residents of PPR Seri Pantai who are a link between the local government bodies and the PPR residents. Due to the current COVID-19 lockdown, face-to-face interviews were not possible. Hence, an online questionnaire using Google forms was designed, which was disseminated by the community leaders to all eligible participants. Eligible participants were all residents of PPR Seri Pantai aged ≥ 18 years of age, and we included all of those who were willing to participate. We employed a convenience sampling technique to include as many eligible residents as possible to participate in the survey. This was essential due to the ongoing pandemic as access to the community was not possible due to the imposed lockdown. To improve participation, the community leaders informed the community about the purpose of the survey and a convenient time for completing the data collection form was ascertained. The community leaders informed the residents about the need to read through and understand the consent form provided alongside the questionnaire. After the participant recorded their consent using a tick box, the survey form became available for them to complete. A total of 180 participants completed all elements of the survey.

### Study instrument

We used the Malay version of the WHOQOL-BREF questionnaire to assess the QoL of the respondents and the DASS-21 to study the perceived depression, anxiety, and stress of the respondents. Sociodemographic data, including age, gender, race, educational level, marital status, and occupation were also recorded.

***The WHOQOL-BREF questionnaire***

Harper *et al.* (1998) has validated the 26-item WHOQOL-BREF as a reliable alternative to WHOQOL-100. The WHOQOL-BREF has been demonstrated to be suitable for assessing the perceived QoL of community-dwelling populations. It comprises 26 items, categorised into a physical domain (seven items), a psychological domain (six items), a social relations domain (three items), and an environment domain (eight items). Each item is ranked using a 5-point Likert scale. The 5-point Likert scale measures the level of agreement to a given statement. The points 1, 2, 3, 4 and 5 each corresponds to ‘strongly disagree,’ ‘disagree,’ ‘neither agree nor disagree,’ ‘agree,’ and ‘strongly agree’ respectively. The total scores were transformed into scores out of 100, with 0 being the least favorable QoL and 100 being the most favorable ( Cruz *et al*., 2011; Harper *et al*., 1998). The questionnaire provides a brief, comprehensive, and multilingual measurement of QoL ( Berwick *et al.*, 1991). As a sizable portion of the population were more comfortable using Malay language, a validated Malay version of the multidimensional WHOQOL-BREF questionnaire was adopted ( Cheung *et al*., 2019; Hasanah *et al*., 2003; Skevington *et al*., 2004; Iqbal *et al*., 2020).

***The DASS-21 questionnaire***

The DASS-21 is a 21-item questionnaire used in assessing the level of self-perceived depression, anxiety, and stress in participants aged 14 and above ( Nordin *et al*., 2017). It is the shorter version of the DASS-42 questionnaire and consists of three domains: depression, anxiety, and stress. Each domain is allocated seven questions, with a Likert scale ranging from zero to three ( Lovibond *et al*., 1995). The points on the scale indicate level of agreement to a given statement, with point 0 indicating ‘never,’ 1 ‘sometimes,’ 2 ‘often,’ and 3 ‘almost always.’ The Malay version of this questionnaire was adequately translated with high validity and internal reliability ( Ramli *et al*., 2009). The DASS-21 requires a shorter time to complete compared to DASS-42 and no special training is required ( Musa *et al*., 2007; Oei *et al*., 2013).

### Statistical analysis

The IBM Statistical Package for Social Sciences version 27.0 was used for data analysis. The qualitative variables included age, gender, ethnicity, marital status, educational level and occupation. The quantitative variables included the WHOQOL and DASS-21 scores. Both WHOQOL-BREF and DASS-21 scores were expressed as mean ± standard deviation. Pearson’s correlation was used to compare the scores of the DASS-21 and WHOQOL domains. A p value ≤ 0.05 was considered statistically significant. There were no participants with missing data as all questions in the online form were compulsory.

### Ethics statement

This study received Institutional Review Board approval from Taylor’s University Center for Research Management (HEC 2019/058). All participants were provided with detailed information about the purpose of the study. Written informed consent was obtained from the participants before completion of the online questionnaire. The consent was obtained for participation as well as for the publication of the survey data. Information collected from participants was treated in confidence, and the anonymity of the participants was maintained throughout.

## Results

### Sociodemographic characteristics

A total of 180 individuals participated in the study (see *Underlying data* ( Patil, 2021)). The average age of respondents was 42.1 ± 11.4. The majority of the respondents were females (82.2%), more than 80% of them were Malays, 62.2% were married, 78.3% had secondary level education, and 46.1% were working ( Table 1).

**Table 1.** Sociodemographic characteristics of the respondents (n = 180).

Variable	Frequency (n)	Percentage (%)
Age (mean ± SD) 18 − <40 years ≥40 years	42.1 73 107	11.4 40.6 59.4
Gender Male Female	32 148	17.8% 82.2%
Ethnicity Malay Indian	157 23	87.2 12.8
Marital status Single Married Separated/Divorced/Widowed	33 112 35	18.3 62.2 19.4
Level of education Primary/None Secondary Tertiary	12 141 27	6.7 78.3 15.0
Occupation Working Not working/Student Housewife	83 30 67	46.1 16.7 36.2

### Summary of WHOQOL and DASS-21 scores

Table 2 shows the summary of the scores obtained in the four domains of QoL and the depression, anxiety, and stress subscales. The average scores for the four QoL domains were 66.36 ± 14.27 (physical), 66.96 ± 15.56 (psychological), 64.52 ± 20.38 (social) and 59.28 ± 17.23 (environment). The psychological domain showed the highest scores while the opposite was observed in the environment domain. The DASS-21 scores were highest for stress (5.66 ± 4.59), followed by anxiety (4.91 ± 4.35) and depression (4.66 ± 4.60).

**Table 2.** Distribution of scores of the World Health Organization Quality of Life (WHOQOL) domains and the depression, anxiety, and stress scale (DASS-21) subscales.

Domain Subscale	Mean ± SD	Minimum	Maximum	Skewness	Kurtosis
**WHOQOL Domains**					
Physical	66.36 ± 14.27	25.00	100.00	0.027	−0.493
Psychological	**66.96 ± 15.56**	19.00	100.00	−0.213	−0.202
Social	64.52 ± 20.38	19.00	100.00	−0.015	−0.670
Environment	**59.28 ± 17.23**	29.00	100.00	0.240	0.375
**DASS – 21 Subscales**					
Depression	4.66 ± 4.60	0.00	18.00	0.873	−0.339
Anxiety	4.91 ± 4.35	0.00	18.00	0.834	−0.063
Stress	**5.66 ± 4.59**	0.00	18.00	0.700	−0.374

### Correlation between domains of the WHOQOL and DASS-21 scores

Statistically significant negative associations were observed between all subscales of DASS-21 and all domains of QoL ( Table 3) with the exception of the environment domain. The correlation between the QoL domains and DASS subscales were highly significant (p < 0.001).

**Table 3.** Correlation between scores of the World Health Organization Quality of Life (WHOQOL) domains and the depression, anxiety, and stress scale (DASS-21) subscales.

WHOQOL Domains	DASS-21 subscales					
	Depression (r)	p value	Anxiety (r)	p value	Stress (r)	p value
Physical	−0.294	<0.001	−0.330	<0.001	−0.274	<0.001
Psychological	**−0.520**	<0.001	**−0.446**	<0.001	**−0.496**	<0.001
Social	**−0.448**	<0.001	−0.388	<0.001	**−0.427**	<0.001
Environment	−0.218	0.003	−0.202	0.007	−0.209	0.005

**Figure 1.**
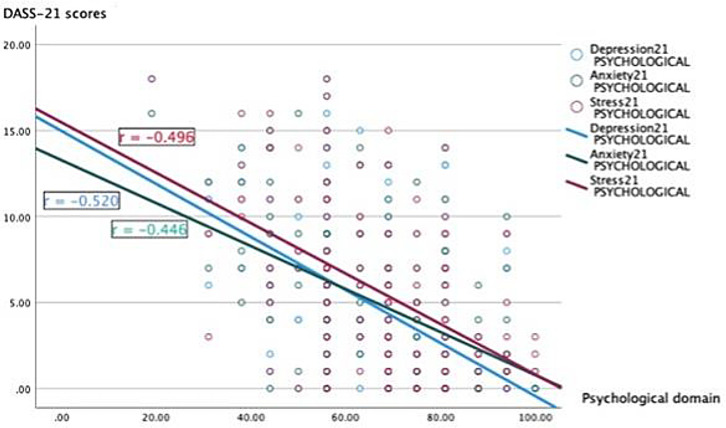
Correlation between the psychological domain scores of Quality of Life and the depression, anxiety, and stress scores.

As shown in Figure 1, the strongest correlation was observed between depression and the psychological domain (r = −0.520, p < 0.001) followed by stress and psychological domain (r = −0.496, p < 0.001).

**Figure 2.**
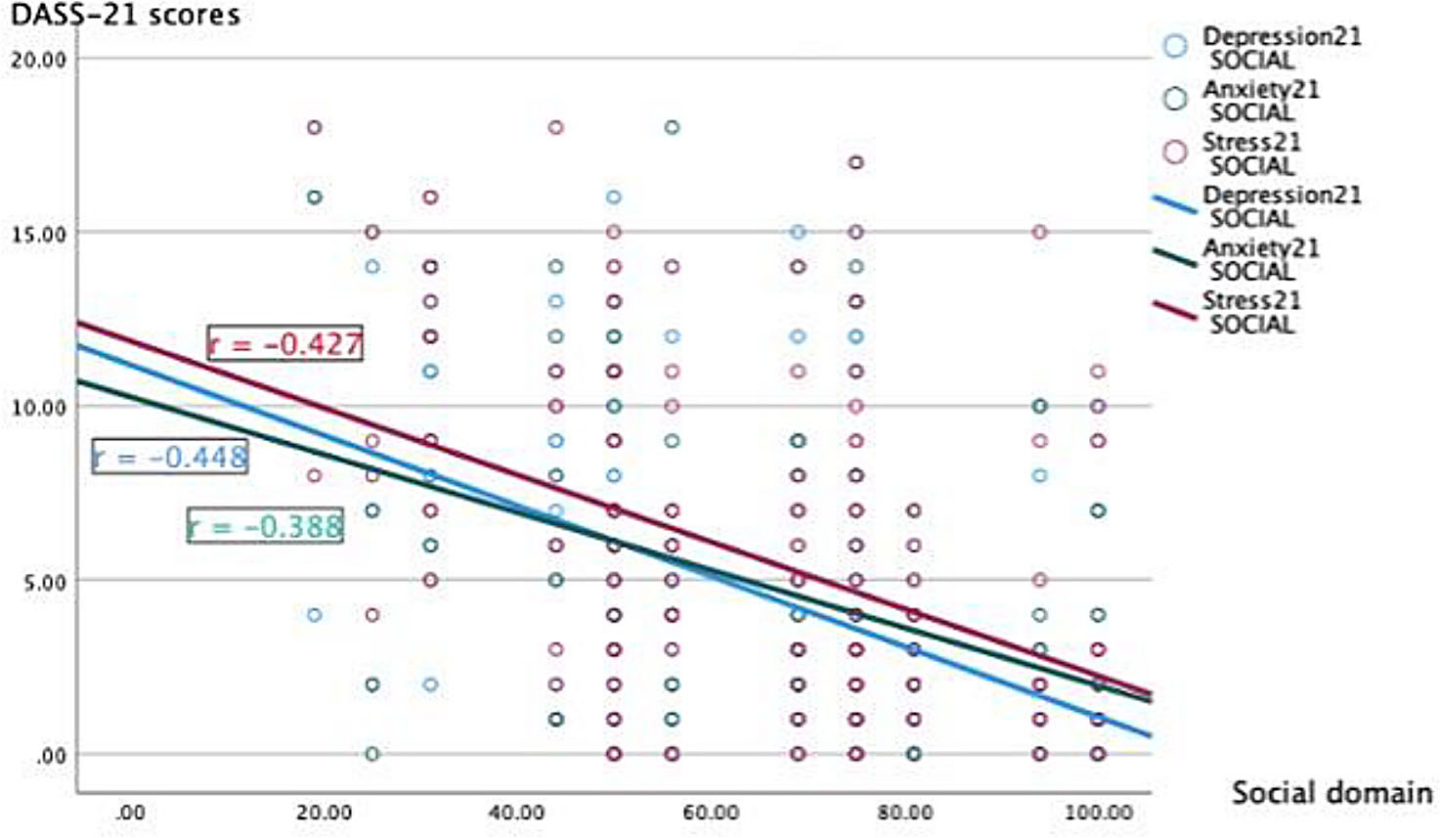
Correlation between the social domain scores of Quality of Life and the depression, anxiety, and stress scores.

The scores for depression showed a strong negative correlation in comparison with the social domain scores (r = -0.448, p < 0.001) and the scores for stress had a correlation coefficient of −0.427, p < 0.001) ( Figure 2).

## Discussion

Notwithstanding the lack of agreement with previous literature by Gan and Hue (2019), our results have shown that the average score for the DASS-21 was highest for the stress subscale. This is also contrasting with a study by Basudan *et al.* (2017) who described a higher level of anxiety compared to depression and stress among dental students in Saudi Arabia. Baum, Garafalo & Yali (1999) found that low socioeconomic status was reliably associated with chronic stress.

The scores for the environment domain were found to be the lowest, indicating that participants felt they had inadequate physical living conditions. This differed from a similar study by Wan Puteh *et al.* (2019) where poor health status (physical health) was found to be the determinant of low QoL. However, a Malaysian study by Zainal *et al.* (2012) offers a possible explanation for our findings, as it presents evidence that poor housing conditions can negatively affect the quality of life of the urban poor.

There was a statistically negative association between all subscales of DASS-21 and all domains of QoL. This supports another Malaysian study by Bujang *et al.* (2015) which found that all psychological aspects of DASS-21 have a negative impact on the community's QoL. The highest clinical relevance was observed between the psychological domain and depression. This is expected, as meta-analysis by Lorant *et al.* (2003) found that those in low socioeconomic groups have a higher probability of being depressed. This resonated with findings reported by Gan and Hue (2019) where depression was significantly associated with psychological domains.

Ettman *et al.* (2020) reported a three-fold increase in depression prevalence during the pandemic compared to before. This could be explained by the ground-to-halt economy, restricted social activities, compromised health, sleep disturbances and disruption to normal routine ( Bignardi *et al.*, 2020; Fountoulakis *et al.*, 2021; Gaidhane *et al.*, 2020; Majumdar *et al.*, 2020; Rehman *et al.*, 2021). Furthermore, social isolation due to lockdown is another risk factor for mental health issues ( Banerjee & Rai, 2020). In addition, the robust causal relationship between life’s stressful events and major depressive episodes further explains the high stress scores during the pandemic lockdown ( Breslau & Davis, 1986; Hammen, 2005; Tafet & Nemeroff, 2016).

## Conclusions

This study found that there is a significant negative association between depression, anxiety, stress and the perceived QoL with the strongest correlation being observed between depression and the psychological domain. Clinical relevance was also found between psychological and social domains of QoL with all of the DASS-21 subscales. This warrants further investigation to determine the causation and nature of this relationship in the context of B40 communities. In addition, the scope of this study needs to be widened to cover other B40 communities in other parts of the country to understand the pattern of association between QoL and the mental health subscales. Similarities and differences in the patterns could assist in planning strategies and measures to address potential associations. Undeniably, the COVID-19 lockdown has left a significant mark on the QoL and mental health status of individuals, adding further stressors to an already disadvantaged and vulnerable population. Therefore, poor QoL and the potentially devastating mental health consequences associated with the pandemic need to be addressed. Further research will pave the way for interventions to be proposed and executed effectively, and these interventions should be replicable in all B40 communities. Vulnerable populations require particular focus so that sustainable change can be implemented to help them through this uncertain season and beyond.

## Limitations

The survey could not be done face-to-face due to COVID-19 lockdown restrictions. The sample size was small and the findings cannot be generalized to all of the B40 communities in Malaysia. Additionally, due to the cross-sectional nature of our study, the causal relationship between QoL and depression, anxiety and stress could not be established. There was an unequal distribution of sociodemographic factors whereby the majority of the participants were females (more than 80%) and Malays (87.2%).

## Data availability

### Underlying data

Harvard Dataverse: PPR Seri Pantai_ Association between QoL and mental health. https://doi.org/10.7910/DVN/NKPXYL ( Patil, 2021).

This project contains the following underlying data:

- PPR Data_final_160321.ods (PPR survey data).
- PPR DATA SYNTAX.sps. (syntax for data analysis).

Data are available under the terms of the Creative Commons Zero “No rights reserved” data waiver (CC0 1.0 Public domain dedication).

## Contributions

Gan Sing Joo

Roles: Conceptualization, Data Curation, Formal Analysis, Investigation, Methodology, Project Administration, Resources, Software, Supervision, Writing – Original Draft Preparation, Writing – Review & Editing.

Daniel Mahalingam Owen Devan

Roles: Investigation, Methodology, Resources, Supervision, Formal Analysis, Writing – Original Draft Preparation, Writing – Review & Editing.

Chuah Shao Qi

Roles: Investigation, Methodology, Resources, Supervision, Writing – Original Draft Preparation, Writing – Review & Editing.

Sapna Shridhar Patil

Roles: Conceptualization, Project Administration, Funding Acquisition, Resources, Software, Supervision, Validation, Visualization, Data Curation, Writing – Original Draft Preparation, Writing – Review & Editing.
