# Supplementary material for: Association between depression, anxiety, stress and perceived quality of life in a Malaysian B40 urban community during the COVID-19 lockdown: A cross-sectional study
Source: F1000Res. 2021 Jul 29;10:693. [Version 1] doi: 10.12688/f1000research.51924.1 (PMC10918314; doi:10.12688/f1000research.51924.1)
Supplement: Peer review history

# Reviewer report: Reviewer response for version 1

**DOI:** 10.5256/f1000research.55136.r251391

**Published:** 2024-03-06

**Authors:** Dr.Richard Mottershead

**Affiliations:** 1 University of Sharjah, Sharjah, Sharjah, United Arab Emirates

Firstly, I would like to thank the authors for allowing me to review their study on the association between QoL and mental health in an underprivileged urban community in Malaysia during the period of mobility restrictions in the covid 19 pandemic. 

 The study provides an important insight into an underprivileged community and their lived experiences during the recent pandemic. The impact on QoL during these restrictions is of importance to provide understanding for future humanitarian crisis. The study also allows for comparisons with other ethnographic groups creating a wider perspective. 

 This is a well-composed and structured article. The authors appear to have a creditable background and research experience which gives the article credibility. Comparisons have been made with relevant contemporary research and this allows for a global overview of the topic and how this article fits within the body of evidence.  I think that the authors are correct not to generalize and to highlight the subjectivity of examining one sample within a Malaysian urban community. However, the data does provide useful insight and knowledge into the lived experience of those experiencing humanitarian challenges.  

 The methodology and methods are clear and would allow for the study to be replicated. Ethical approval is clearly listed as is the data collection technique used and provides transparency to the reader. 

 The conclusion is clear, concise and informative with recommendations that would suggest areas for future research.

Is the work clearly and accurately presented and does it cite the current literature?

Yes

If applicable, is the statistical analysis and its interpretation appropriate?

Yes

Are all the source data underlying the results available to ensure full reproducibility?

Yes

Is the study design appropriate and is the work technically sound?

Yes

Are the conclusions drawn adequately supported by the results?

Yes

Are sufficient details of methods and analysis provided to allow replication by others?

Yes

Reviewer Expertise:

Life story and narrative research. Post-crisis research, emancipatory research, psychiatric and mental health research.

I confirm that I have read this submission and believe that I have an appropriate level of expertise to confirm that it is of an acceptable scientific standard.

# Reviewer report: Reviewer response for version 1

**DOI:** 10.5256/f1000research.55136.r122017

**Published:** 2022-02-15

**Authors:** Gajanan Dattatray Velhal

**Affiliations:** 1 Community Medicine, Seth G S Medical College and KEM Hospital, Mumbai, Maharashtra, India

This article has attempted to study the association between quality of life and mental health in an underprivileged urban community in Malaysia during the period of mobility restrictions imposed to curb COVID-19 transmission.

 The authors have attempted to explore an extremely relevant topic in the wake of the current situation with imposed restrictions, loss of financial security, and the need to sustain and survive during a crisis that is both medical and humanitarian in nature.

 Although the study has been performed only in a single B40 community, the results justify the need to conduct such studies in several such communities to assess the extent of the problem and address the emerging issues. The authors possibly could look into the causal relationship between mental health and quality of life by adopting  analytical type of study design for a better insight.

- Is the work clearly and accurately presented and does it cite the current literature? The work has been presented in an understandable manner, the language is easy to comprehend and the literature review conforms to the requirement of being recent.
- Is the study design appropriate and does the work have academic merit?  For the given objective, the design of the study looks appropriate. The work of the authors highlights the need to address significant issue affecting health and well-being.
- Are sufficient details of methods and analysis provided to allow replication by others? The methodology used for this study has been detailed out clearly and the analysis is aligned to the objectives. The survey instruments used have been described in adequate detail and the data collection technique has been clearly described.
- If applicable, is the statistical analysis and its interpretation appropriate?  The statistical analysis is clearly mentioned  and the results reflect the performed analysis. The interpretation of the obtained results is in line with the requirements.
- Are all the source data underlying the results available to ensure full reproducibility? Yes the source data is available.
- Are the conclusions drawn adequately supported by the results? The conclusions are drawn in line with the observations made by the authors. The recommendations made by the authors in the context of the pandemic highlight the further research and strategies required to address the problem.

Is the work clearly and accurately presented and does it cite the current literature?

Yes

If applicable, is the statistical analysis and its interpretation appropriate?

Yes

Are all the source data underlying the results available to ensure full reproducibility?

Yes

Is the study design appropriate and is the work technically sound?

Yes

Are the conclusions drawn adequately supported by the results?

Yes

Are sufficient details of methods and analysis provided to allow replication by others?

Yes

Reviewer Expertise:

Epidemiology, Communicable & Non communicable diseases, Nutrition, Health Planning

I confirm that I have read this submission and believe that I have an appropriate level of expertise to confirm that it is of an acceptable scientific standard.
